# Interleukin-18 Down-Regulates Multidrug Resistance-Associated Protein 2 Expression through Farnesoid X Receptor Associated with Nuclear Factor Kappa B and Yin Yang 1 in Human Hepatoma HepG2 Cells

**DOI:** 10.1371/journal.pone.0136215

**Published:** 2015-08-20

**Authors:** Xiao-cong Liu, Wei Lian, Liang-jun Zhang, Xin-chan Feng, Yu Gao, Shao-xue Li, Chang Liu, Ying Cheng, Long Yang, Xiao-Juan Wang, Lei Chen, Rong-quan Wang, Jin Chai, Wen-sheng Chen

**Affiliations:** 1 Department of Gastroenterology, Southwest Hospital, Third Military Medical University, Chongqing, People’s Republic of China; 2 Department of Gastroenterology, Chengdu Military General Hospital, Chengdu, Sichuan, People’s Republic of China; 3 Department of Burn and Plastic Surgery, Chengdu Military General Hospital, Chengdu, Sichuan, People’s Republic of China; Nihon University School of Medicine, JAPAN

## Abstract

Multidrug resistance-associated protein 2 (MRP2) plays an important role in bile acid metabolism by transporting toxic organic anion conjugates, including conjugated bilirubin, glutathione, sulfate, and multifarious drugs. MRP2 expression is reduced in cholestatic patients and rodents. However, the molecular mechanism of MRP2 down-regulation remains elusive. In this report, we treated human hepatoma HepG2 cells with interleukin-18 (IL-18) and measured the expression of MRP2, nuclear factor kappa B (NF-κB), farnesoid X receptor (FXR), and the transcription factor Yin Yang 1 (YY1) by quantitative real-time quantitative polymerase chain reaction (PCR) and western blotting. We found that expression of MRP2 was repressed by IL-18 at both the mRNA and protein levels in a dose- and time-dependent manner. Furthermore, the activated NF-κB pathway increased YY1 and reduced FXR. These changes were all attenuated in HepG2 cells with knockdown of the NF-κB subunit, p65. The reduced expression of FXR and MRP2 in HepG2 cells that had been caused by IL-18 treatment was also attenuated by YY1 knockdown. We further observed significantly elevated IL-18, NF-κB, and YY1 expression and decreased FXR and MRP2 expression in bile duct-ligated Sprague Dawley rat livers. Chromatin immunoprecipitation assays also showed that FXR bound to the promoter region in MRP2 was less abundant in liver extracts from bile duct-ligated rats than sham-operated rats. Our findings indicate that IL-18 down-regulates MRP2 expression through the nuclear receptor FXR in HepG2 cells, and may be mediated by NF-κB and YY1.

## Introduction

Multidrug resistance-associated protein 2 (MRP2, ABC subfamily C [ABCC]2/Abcc2) is a member of the adenosine triphosphate-binding cassette (ABC) transporter superfamily that is primarily located on the apical membrane (i.e., canalicular membrane) of hepatocytes, renal proximal tubules, and intestines [1, 2]. MRP2 is a conjugate export pump that transports a wide range of compounds, in particular, conjugates of lipophilic substances with glutathione, glucuronate, and sulfate [1, 3] and multifarious drugs such as eprosartan[4] and cisplatin [5]. MRP2 is the major excretion pathway of glucuronidated bilirubin from hepatocytes to the biliary ducts. Mutations and genetic variants of MRP2 in humans may lead to Dubin-Johnson syndrome [6], increased susceptibility for non-alcoholic fatty liver disease [7], and increased risk of bile duct cancer [8]. However, hepatic MRP2 expression is impaired in jaundiced patients with severe alcoholic hepatitis [9] and patients with obstructive cholestasis [10, 11]. In spite of these findings, the precise regulation mechanism of MRP2 in cholestasis remains unknown.

Hepatic cholestasis is always accompanied by various inflammatory cytokines that develop the defensive mechanisms of the damaged liver [12]. Many studies have confirmed the hypothesis that cytokines, especially tumor necrosis factor (TNF)-α, interleukin (IL)-6, and IL-1β, are essential to the regulation of transporter expression in cholestasis; however, the understanding of the mechanism by which other cytokines regulate transporter expression remains unclear [13–16]. IL-18, a member of the IL-1 cytokine family, was also reported to be obviously elevated in patients with obstructive jaundice, and returned to normal levels after the obstruction was relieved [17]. Furthermore, IL-18 was considered to be associated with the depression of hepatic MRP2 in necrotic enteritis in rodents [18]. These findings indicate that increased IL-18 may not only be a bodily response to cholestasis, but may also have effects on the development of cholestasis, specifically the suppression of MRP2 expression in the liver. The aim of this study is to determine the role of IL-18 in the regulation of MRP2.

## Materials and Methods

### HepG2 cells culture and treatment

Human hepatoma HepG2 [19] cells were purchased from American Type Culture Collection, USA (Catalogue No. ATCC HB-8065). HepG2 cells were normally cultured with minimum essential medium (MEM) containing 10% fetal bovine serum (FBS). Before chemical treatment, HepG2 cells were cultured with MEM containing 2% charcoal-stripped FBS (Biological Industries, Kibbutz Beit Haemek, Israel) for at least 12 h, and then treated with recombinant human IL-18 (BioVision Incorporated, Milpitas, USA).

### Animal studies

Ten male Sprague Dawley (SD) rats, aged seven to eight weeks and weighing between 250 and 300 g, were purchased from the Center of Laboratory Animals, Institute of Field Surgery, Daping Hospital, Third Military Medical University, Chongqing, China. All rats were individually housed in plastic cages at 20–23°C, with humidity ranging between 40% and 60% and light cycles set to 12-hour light and 12-hour dark. All rats were fed a standard diet of rodent pellets and purified water. Before starting the experiments, the rats were allowed seven days to adapt to a new environment. All 10 rats were anaesthetized with an intraperitoneal (i.p.) injection of chloral hydrate (300 mg/Kg), and then randomly divided into two groups: the bile duct-ligated (BDL) group (n = 5) and the sham operation group (n = 5). In the BDL group, a midline abdominal incision was made to every rat and the common bile duct (CBD) was doubly ligated with 4–0 silk sutures. In the sham operation group, after the midline abdominal incision, the CBD was exposed and a 4–0 silk suture was passed through, below the CBD. Abdominal closure was performed with a running suture that used 3–0 silk suture for all rats. After the surgical operation, all rats were fed ibuprofen (72 mg/Kg) for three days to relieve their pain. All rats were euthanized with an i.p. injection of sodium pentobarbital (50 mg/Kg), 7 days post-surgery, and their livers were collected for RNA and protein extraction. The animal protocol was reviewed and approved by the Ethics Committee of the Third Military Medical University, Chongqing, China. All sections of this report adhere to the ARRIVE Guidelines for reporting animal research [20]. A completed ARRIVE guidelines checklist is included in S1 Table.

### RNA interference in HepG2 cells

HepG2 cells were transfected with short hairpin RNA (shRNA) targeting the NF-κB subunit p65 or Yin Yang 1 (YY1; Shanghai Genechem Co. Ltd, China) using the X-tremeGENE HP DNA Transfection Reagent (Roche, Mannheim, Germany) for 24 h. Stably transfected cells were selected for with puromycin.

### RNA extraction and quantitative real-time PCR (qPCR)

The total RNA from cultured HepG2 cells and rat livers was extracted with the Trizol reagent (Invitrogen, San Diego, CA) and reverse transcribed into complementary DNA (cDNA) using the PrimeScript RT reagent Kit with gDNA Eraser (Takara Biotechnology, Tokyo, Japan). The qPCR was performed in the Bio-Rad CFX96 real-time PCR detection system (Bio-Rad, Hercules, CA), using the SYBR Premix Ex Taq II kit (Takara Biotechnology). Primers for specific genes are listed in Table 1. Glyceraldehyde 3-phosphate dehydrogenase (GAPDH) was used as a reference for normalizing data.

**Table 1:** Primers Used for qPCR.

Gene	Species*	Sense Primer (5’→3’)	Antisense Primer (5’→3’)
MRP2	H	ctcacttcagcgagaccg	ccagccagttcagggttt
	R	acctccatcaccctcttcaata	aatcgtctcctcccaaatacct
p65	H	gggaaggaacgctgtcagag	tagcctcagggtactccatca
	R	gacctggagcaagccattag	cactgtcacctggaagcaga
YY1	H	ggaatacctggcattgacc	tctttgtgcagcctttgag
	R	tacctggcattgacctctca	ggtgtgcagatgctttctca
FXR	H	cctcctcacctcattgtctc	acctgccacttgttctgtta
	R	tgagcgtctacagcgaaagtg	gggatggtggtcttcaaataag
IL-18	R	atatcgaccgaacagccaac	ttccatccttcacagataggg
GAPDH	H	accacagtccatgccatcac	tccaccaccctgttgctgta
	R	acagcaacagggtggtggac	tttgagggtgcagcgaactt

H: human; R: rat.

### Protein extraction and Western blot analysis

Total and nuclear protein from HepG2 cells and rat livers were extracted using the Total Protein Extraction Kit and the Nuclear Protein Extraction Kit, respectively (Keygen Biotech, Nanjing, China). Protein concentrations were determined with the Pierce BCA Protein Assay Kit (Pierce Biotechnology, Rockford, USA). The dilution of primary antibodies were as follows: rabbit polyclonal to MRP2 (1:1600; Lifespan Biosciences, Seattle, USA), rabbit monoclonal to FXR (1:10000; Epitomics, Burlingame, USA), mouse polyclonal to NF-κB p65 subunit (1:1000), rabbit polyclonal to constitutively acting receptor (CAR; 1:1000), mouse monoclonal to pregnane X receptor (PXR; 1:1000), rabbit polyclonal to retinoic acid receptor α (RARα; 1:1000), rabbit polyclonal to retinoid X receptor α (RXRα; 1:1000), rabbit polyclonal to cholesterol 7-alpha hydroxylase (Cyp7a1) (1:800), Src-homology 2 (SH2)-containing protein tyrosine phosphatases 1 (SH-PTP1; 1:1000), rabbit polyclonal to GAPDH (1:1000; Santa Cruz Biotechnology, Santa Cruz, USA), rabbit monoclonal to phosphorylated NF-κB subunit p65 (pS536; 1:10000; Epitomics), and rabbit polyclonal to YY1 (1:1000; Proteintech Group, Chicago, USA). GAPDH and SH-PTP1 were used as loading references to calibrate the target protein signals for data analysis.

### Chromatin immunoprecipitation assays

Chromatin immunoprecipitation (ChIP) assays were performed using the ChIP Assay Kit (Millipore, Billerica, USA). Rat liver tissues were homogenised and fixed with 1% formaldehyde. DNA was sheared to 200–1000 bp fragments using sonication. Chromatin was incubated and precipitated with antibodies against FXR, histone deacetylase 3 (HDAC3; Epitomics), or IgG (Boster, Wuhan, China). Primers used to amplify the binding site of FXR on the promoter region of MRP2 gene for qPCR were designed as follows: sense- 5’-agagtgagttccaggacagt-3’; antisense- 5’-ccctgtagtgtgaccaaata-3’.

### Statistical Analysis

Statistical analyses were performed using the Statistical Package for Social Science software, version 19.0 (SPSS, Chicago, USA). Data from the two groups were analyzed using a two-tailed independent *t*-test, where *P*<0.05 was considered significant.

## Results

### MRP2 expression in HepG2 cells is repressed by IL-18

**Fig 1:**
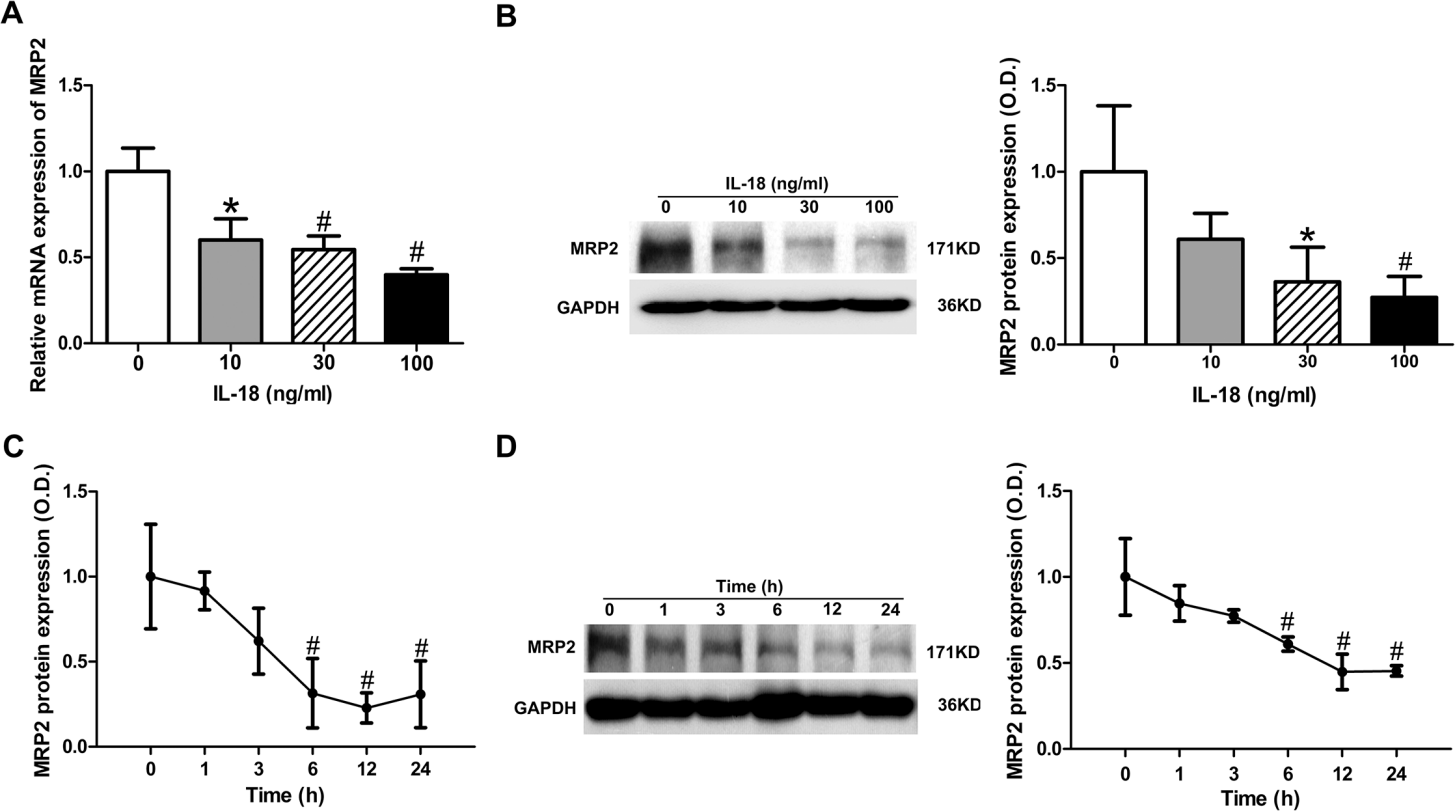
IL-18 repressed MRP2 expression in HepG2 Cells. Different doses of IL-18 (i.e., 0, 10, 30, 100 ng/ml) repressed MRP2 expression in HepG2 cells by SYBR green qPCR (A) and western blot analysis (B). IL-18 (30 ng/ml) repressed MRP2 expression in a time-dependent manner in HepG2 cells by SYBR green qPCR (C) and western blot analysis (D). Data is given as the mean ± SD (n = 3). *P<0.05.vs. baseline; ^#^ *P*<0.01 vs. baseline.

To test whether IL-18 influenced the expression of MRP2, we treated cultured HepG2 cells with IL-18 at different doses. We first quantified the mRNA levels of MRP2 by qPCR. Down-regulation of MRP2 at the mRNA level was observed (Fig 1A). We detected the protein level of MRP2 by western blotting and down-regulation was also observed (Fig 1B). Doses of IL-18 over a range of 10–100 ng/ml were used for treatment, and the results indicated a dose-dependent inhibition in both mRNA and protein levels (Fig 1A and 1B). To gain further insight into the regulated effect, we then examined the time-dependent effect of IL-18 on MRP2 on both mRNA and protein levels. IL-18 (30 ng/ml) produced a decreased expression of MRP2 as early as 1 h and reached a maximal effect at 12 h for both mRNA and protein levels (Fig 1C and 1D). At 24 h, an increased expression of MRP2 mRNA levels was observed; however, the decreased expression of MRP2 protein levels was sustained up until 24 h. Taken together, these results suggest that expression of MRP2 was repressed by IL-18 in a dose- and time-dependent manner, at both the mRNA level and protein level.

### IL-18 repressed FXR mRNA and protein levels

**Fig 2:**
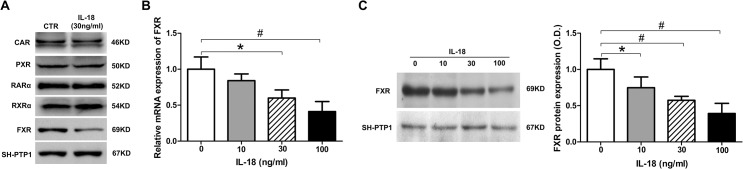
IL-18 represses FXR mRNA and protein levels. Expression of CAR, PXR, RARα, RXRα, and FXR in normal and IL-18-treated HepG2 cells by western blot analysis (A). IL-18 repressed FXR mRNA levels in a dose-dependent manner in HepG2 cells by SYBR green qPCR analysis (B). IL-18 repressed FXR protein expression in a dose-dependent manner in HepG2 cells by western blot analysis (C). Data is given as the mean ± SD (n = 3). *P<0.05.vs. baseline; ^#^ *P*<0.01 vs. baseline.

MRP2 has been shown to be up-regulated by several nuclear receptors, such as FXR, PXR, CAR [21], and RAR [22]; a decrease in any of these nuclear receptors may lead to reduced MRP2 expression. We detected several nuclear receptors in HepG2 cells treated by IL-18 and found that FXR protein expression declined significantly upon treatment (Fig 2A). To gain further insight into the effect of IL-18 on the expression of FXR, we next examined the dose-dependent effect of IL-18 on FXR mRNA and protein levels. mRNA and protein levels of FXR decreased significantly after IL-18 treatment. Treatment of IL-18 over a dose range of 10–100 ng/ml was showed a dose-dependent regulation of both mRNA and protein (Fig 2B and 2C). These data suggested that decreased levels of FXR might result in reducing the expression of MRP2 via treatment with IL-18.

### IL-18 induced YY1 expression, which repressed FXR and MRP2 expression

**Fig 3:**
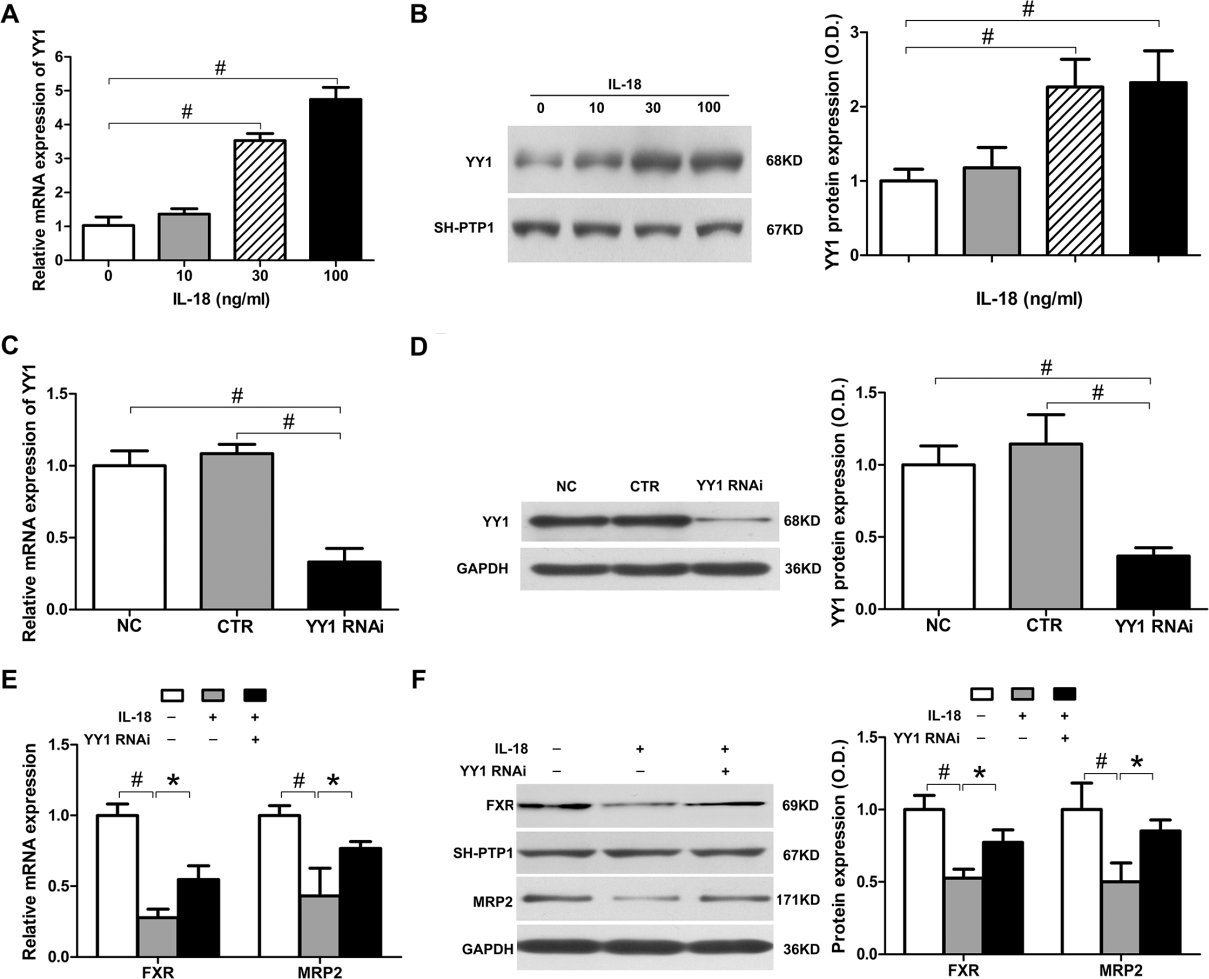
IL-18 induced YY1 expression, which repressed FXR and MRP2 expression. (A-B) IL-18 enhanced YY1 expression in HepG2 at the mRNA level by SYBR green qPCR (A) and at the protein level by western blot analysis (B) in a dose-dependent manner. (C-D) HepG2 cells were transfected with control and YY1 shRNA. YY1 mRNA was measured by SYBR green qPCR (C) and protein was measured by western blot analysis (D). (E-F) HepG2 cells were transfected with YY1 shRNA and then incubated with IL-18 (30 ng/ml) for 24 h. MRP2 and FXR mRNA was measured by SYBR green qPCR (E) and protein expression was measured by western blot analysis (F). Data is given as the mean ± SD (n = 3). *P<0.05, ^#^ *P*<0.01.

A recent study in obese mice demonstrated that FXR can be suppressed by the YY1 transcription factor [23]. To determine whether YY1 was associated with decreased levels of FXR, we first detected the expression of YY1 in IL-18-treated HepG2 cells. Treatment of HepG2 cells with different doses of IL-18 markedly increased the expression of YY1 in both mRNA and protein levels, which demonstrated that IL-18 treatment also affected the expression of YY1 (Fig 3A). To determine whether the YYI-increased expression induced by IL-18 treatment resulted in decreased expression of FXR and MRP2, we next transfected HepG2 cells with YY1 shRNA. The mRNA and protein expression of YY1 was suppressed to 33.1% and 36.9% as compared to the non-treated control (Fig 3B), respectively, after YY1 knockdown. We confirmed that IL-18 inhibited FXR (Fig 2) and MRP2 (Fig 1) expression significantly in normal HepG2 cells. However, FXR and MRP2 expression were only slightly decreased in YY1-interfered HepG2 cells after IL-18 treatment. As showed in Fig 3C, in normal HepG2 cells with IL-18 treatment, FXR and MRP2 mRNA expression were decreased to 27.8% and 43.2% as compared to the non-treated control, respectively. The FXR and MRP2 protein expression decreased to 52.7% and 50.2% as compared to the non-treated control, respectively; however, in YY1 knockdown HepG2 cells with IL-18 treatment, FXR and MRP2 mRNA expression decreased to 54.7% and 76.6% as compared to the non-treated control, respectively, and the protein expression decreased to 77.3% and 85.2%. In other words, the reduction of FXR and MRP2 induced by IL-18 was attenuated by YY1 interference. These data indicate that elevated YY1 induced by IL-18 reduces FXR and MRP2 expression in HepG2 cells.

### NF-κB signaling is activated by IL-18 and mediates the regulation of YY1, FXR, and MRP2

**Fig 4:**
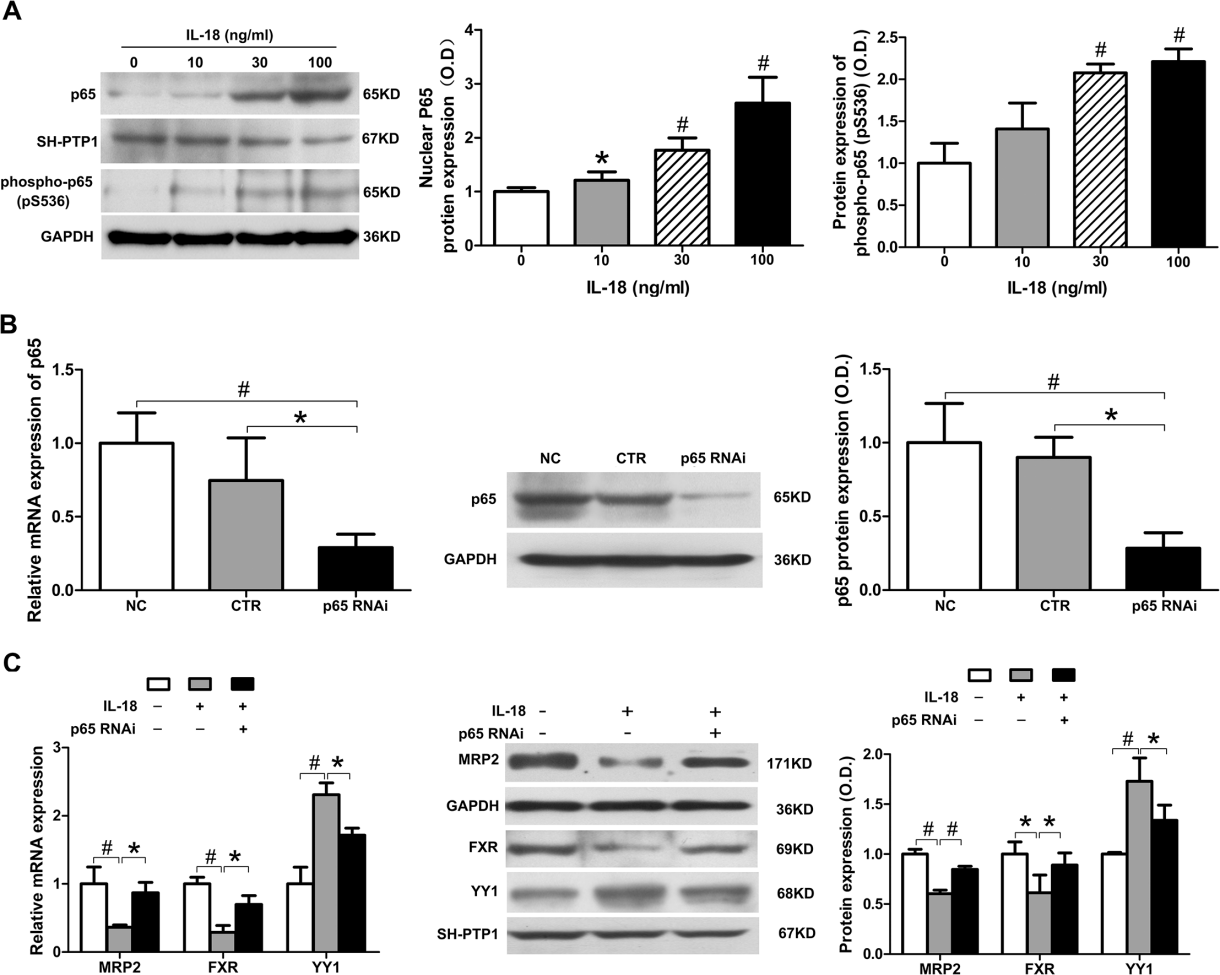
NF-κB signaling is activated by IL-18 and mediates the regulation of YY1, FXR, and MRP2. (A) In HepG2 cells, IL-18 increased levels of p65 in the nucleus and phosphorylated p65 in total protein in a dose-dependent manner, as measured by western blot analysis. (B) HepG2 cells were transfected with control and YY1 shRNA. YY1 expression was measured by SYBR green qPCR and western blot analysis. (C) HepG2 cells were transfected with YY1 shRNA and then incubated with IL-18 (30 ng/ml) for 24 h; MRP2 and FXR expression was then measured by SYBR green qPCR and western blot analysis. Data is given as the mean ± SD (n = 3). *P<0.05, ^#^ *P*<0.01.

YY1 expression is known to be enhanced by NF-κB [24], which is a main signal transduction pathway of IL-18 [25]. NF-κB is a nuclear transcription factor that is crucial for the regulation of inflammation, and is one of the main signaling pathways of IL-18. NF-κB is sequestered in the cytoplasm and inhibits a number of proteins by binding to them. Once activated, the inhibited proteins are ubiquitinated and subsequently degraded, resulting in the phosphorylated p65/p50 hetero-dimer, which is the main form of activated NF-κB. Phosphorylated NF-κB is then translocated to the nucleus and activates the transcription of certain genes. Therefore, we further examined the role of NF-κB in transcriptional regulation of MRP2 by detecting changes in NF-κB expression in isolated nuclear proteins and phosphorylated NF-κB in total cellular protein. We treated HepG2 cells with IL-18 (0, 10, 30, and 100 ng/ml) for 12 h, then extracted both total and nuclear protein. Western blot analysis showed that the amount of total p65 in the nuclear fraction significantly increased after IL-18 treatment, and the phosphorylated p65 in total protein showed a similar trend (Fig 4A). These results suggest that NF-κB was activated by IL-18 in HepG2 cells in a dose-dependent manner.

We next determined whether NF-κB signaling participated in IL-18-induced MRP2 down-regulation. We inhibited the expression of p65 in HepG2 cells by p65 shRNA transfection. The mRNA and protein expression of p65 was suppressed to 29.1% and 28.4% after RNA interference, as compared to the non-treated control, respectively (Fig 4B). We measured the expression of MRP2 in normal HepG2 cells and p65 shRNA-transfected HepG2 cells treated by IL-18 (30 ng/ml). The mRNA and protein level of MRP2 in normal HepG2 cells with IL-18 treatment were decreased to 28.4% and 60.3% as compared to the non-treated control, respectively. In the p65 knockdown cells, the mRNA and protein expression of MRP2 decreased to 77.1% and 74.7% as compared to the non-treated control, respectively (Fig 4C), suggesting that NF-κB is a crucial factor in the down-regulation of MRP2 by IL-18.

We further detected the expression of YY1 and FXR in p65 shRNA-transfected HepG2 cells treated with IL-18. In normal HepG2 cells treated with IL-18, the mRNA and protein expression of YY1 increased 2.3- and 1.7-fold, respectively, but increased only 1.7- and 1.3-fold in the p65 knockdown HepG2 cells (Fig 4C). Meanwhile, the mRNA and protein expression of FXR were reduced to 28.9% and 61.4% as compared to the non-treated control, respectively, in normal HepG2 cells with IL-18 treatment, but were only reduced to 69.8% and 89.1% as compared to the non-treated control, respectively, in p65 knockdown HepG2 cells (Fig 4C). Thus, the alteration of YY1 and FXR expression induced by IL-18 was attenuated in the p65 shRNA-transfected HepG2 cells, at both the mRNA and protein level. These findings demonstrate that IL-18 can up-regulate YY1 expression and down-regulate FXR and MRP2 expression, and that regulation is mediated by the NF-κB signaling pathway.

### Elevated IL-18, NF-κB p65, and YY1 expression, and decreased FXR and MRP2, were observed in bile duct-ligated rats

**Fig 5:**
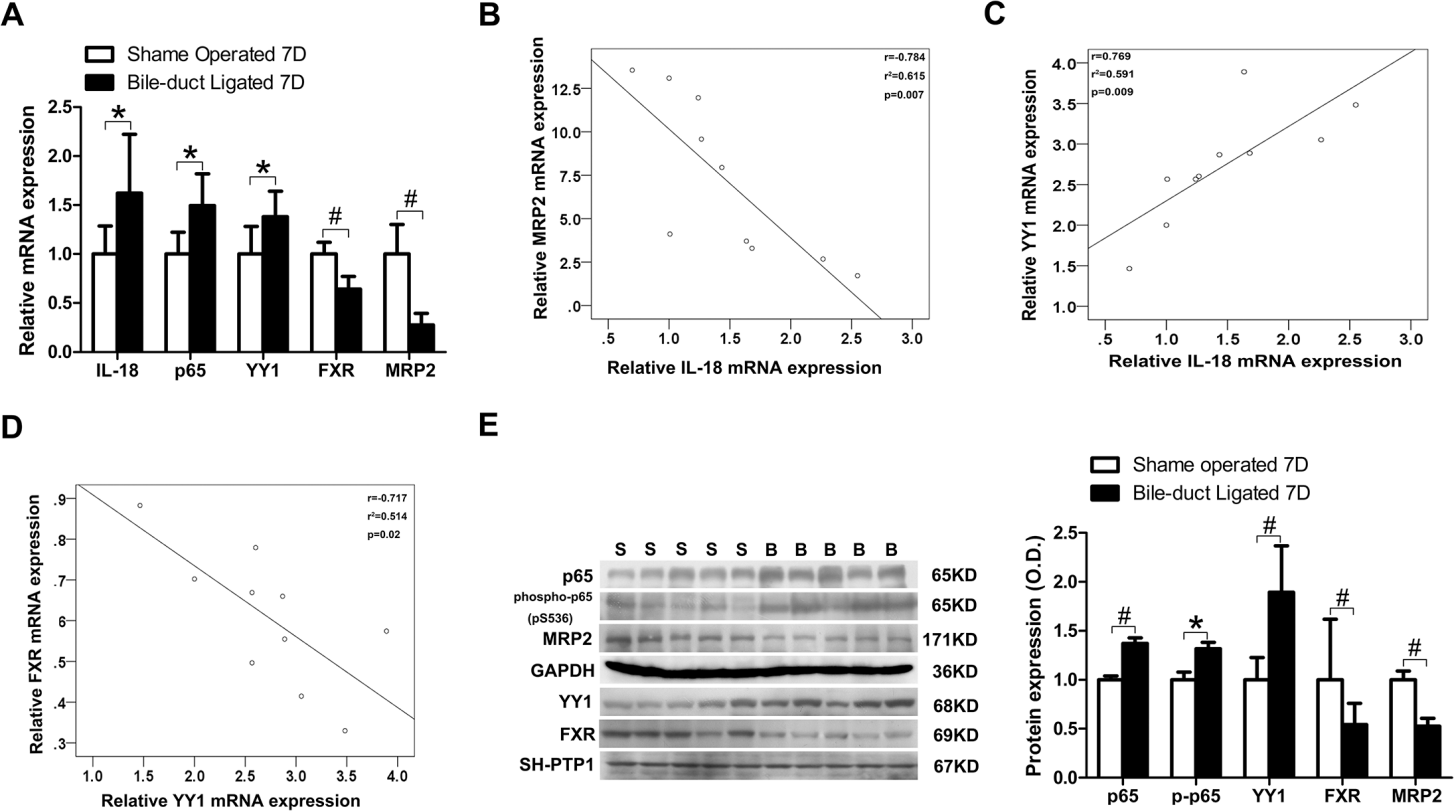
Elevated IL-18, NF-κB p65, and YY1 expression and decreased FXR and MRP2 were observed in the BDL rats. SD rats were divided into the BDL group (n = 5), receiving bile duct ligation, and the sham operation group (n = 5), receiving CBD exposure with a 4–0 silk suture passed through below the CBD. Samples were collected 7 days after surgery. (A) The mRNA levels of IL-18, p65, YY1, FXR, and MRP2; (B) IL-18 mRNA levels are negatively correlated with MRP2 mRNA expression; (C) IL-18 mRNA levels are positively correlated with YY1 mRNA expression; (D) FXR mRNA levels are negatively correlated with YY1 expression; (E) protein levels of p65, phosphorylated p65, YY1, FXR, and MRP2. S, sham operation group; B, bile duct-ligated group; *P<0.05, ^#^ *P*<0.01.

We collected liver samples from sham-operated rats and bile duct-ligated (BDL) rats, 7 days post-surgery, then measured the expression of IL-18, p65, YY1, FXR, and MRP2 in the livers. We observed that the mRNA expression of IL-18, p65, and YY1 in the BDL group were significantly increased (1.6-, 1.5- and 1.4-fold, respectively) when compared to the sham-operated group, whereas FXR and MRP2 mRNA expression were significantly deceased to 64% and 28% of the sham-operated group, respectively (Fig 5A). We analyzed the mRNA expression of these genes by linear regression to examine whether any correlation existed between these genes. Analyses demonstrated that IL-18 mRNA levels were significantly negatively correlated with MRP2 mRNA levels (*r* = 0.784, *p* = 0.007) but positively correlated with YY1 mRNA levels (*r* = 0.769, *p* = 0.009; Fig 5B and 5C). Meanwhile, YY1 mRNA levels were significantly negatively correlated with FXR mRNA levels (*r* = 0.717, *p* = 0.02; Fig 5D). We further detected protein expression by western blotting, which revealed that p65, phosphorylated p65, and YY1 protein expression in the BDL group were increased (1.4-, 1.3- and 1.9-fold, respectively) when compared to the sham-operated group, while FXR and MRP2 protein levels in BDL group decreased to 54% and 52% of the sham-operated group, respectively (Fig 5E).

### FXR bound to the promoter region on MRP2 was reduced in bile duct-ligated rats

**Fig 6:**
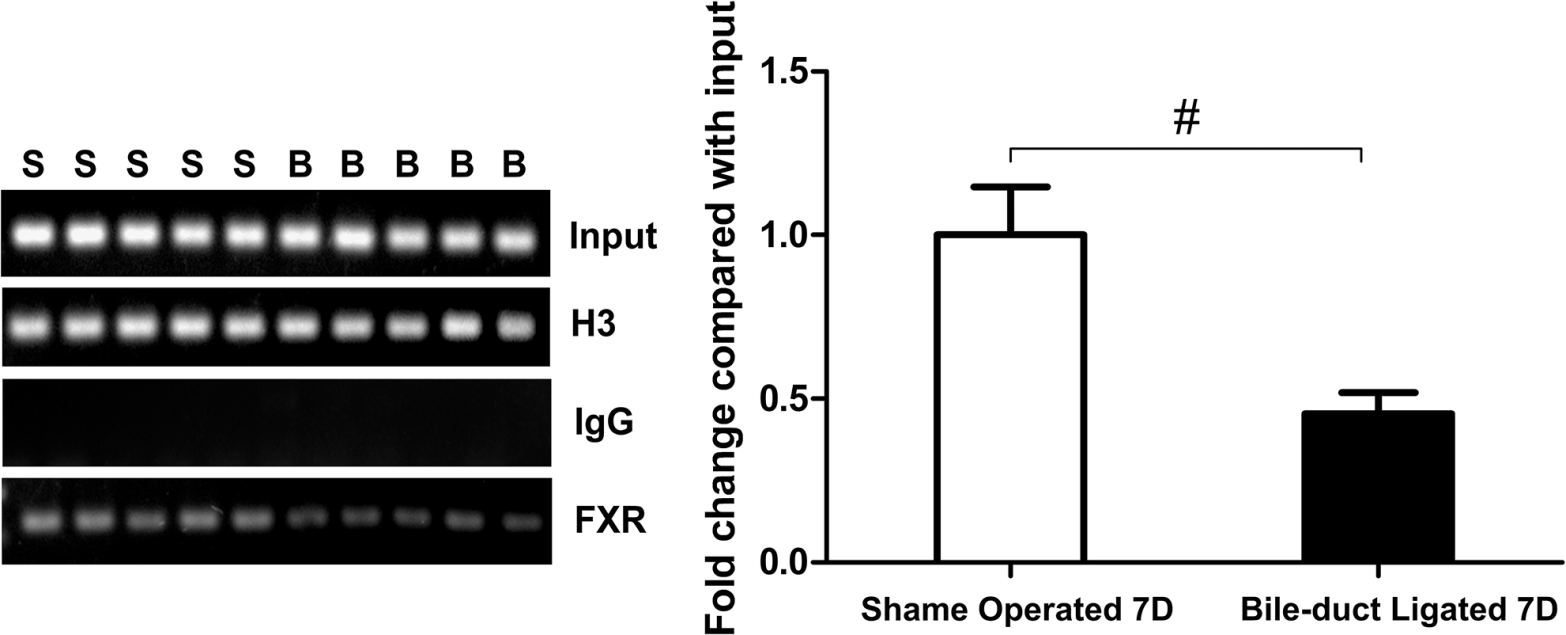
FXR bound to the promoter region on MRP2 was reduced in the BDL rats. SD rats were divided into the BDL group (n = 5), receiving bile duct ligation, and the sham operation group (n = 5), receiving CBD exposure with a 4–0 silk suture passed through below the CBD. Samples were collected 7 days after surgery. ChIP assays showed the amount of FXR bound to the MRP2 promoter was significantly less in the BDL rats than sham-operated rats. S, sham operation group; B, bile duct-ligated group; ^#^ *P*<0.01.

FXR has been confirmed to be bound to a 26 bp sequence, 440 bp upstream of the MRP2 transcription initiation site [21]. We collected liver samples from the BDL and sham-operated rats, 7 days post-surgery, and then performed a ChIP assay to determine whether there was a change in the abundance of FXR bound to the promoter in the MRP2 gene. The results showed that FXR bound to the promoter region in MRP2 was less abundant in the liver extracts from BDL rats than sham-operated rats (*P*<0.01; Fig 6). These data indicate that the reduction of MRP2 in the BDL rats is likely due to the reduction of FXR.

## Discussion

We demonstrated that IL-18 can repress MRP2 expression through FXR. Furthermore, we observed that the transcription factor YY1 was enhanced by IL-18 via the NF-κB signaling pathway, and that the decreased expression of FXR was associated with enhanced YY1. These findings may be due to (1) MRP2 and FXR repression and YY1 enhancement by IL-18 in HepG2 cells at both the mRNA and protein level (Figs 1, 2, 3A, and 3B); (2) IL-18 activation of the NF-κB signaling pathway indicated by nuclear p65 and total phosphorylated p65 enhancement (Fig 4A); (3) changes in MRP2, FXR, and YY1 expression induced by IL-18 which were attenuated by p65 knockdown (Fig 4C); (4) attenuation of the IL-18-induced decreases in MRP2 and FXR expression by YY1 knockdown (Fig 3E and 3F); (5) elevated expression of IL-18, p65, and YY1 and decreased expression of FXR and MRP2 observed in the BDL rats (Fig 5A and 5E); and (6) decreased amount of FXR bound to the MRP2 promoter in the BDL rats (Fig 6). These results indicate that IL-18 enhances YY1 expression via NF-κB, leading to the repression of FXR expression by elevated YY1, resulting in reduced of MRP2 expression.

MRP2 is a key transporter for the balance of bile acid metabolism. High expression of MRP2 is critical for the maintenance of enterohepatic bile acid circulation, which may explain why most known molecular regulations of MRP2 are positive. Previous studies have reported that MRP2 expression was down-regulated at both the mRNA and protein level in some pathological states, mainly in cholestasis [26–28]; however, the mechanism for this remains unclear. Many studies have supported the hypothesis that cytokines are essential in the regulation of transporter expression in cholestasis [13]. For example, TNF-α up-regulates MRP3 expression at both the mRNA and protein level [16]. IL-18 was reported to be significantly elevated in patients with obstructive jaundice [17] and other diseases associated with cholestasis, such as primary biliary cirrhosis [29] and intrahepatic cholestasis of pregnancy [30]. Furthermore, IL-18 was associated with the depression of hepatic MRP2 in necrotic enteritis in rodents [18]. These findings indicate that the increased IL-18 maybe not only be a bodily response to cholestasis, but may also affect the development of cholestasis, specifically via the suppression of MRP2 expression in the liver. Our study is the first to confirm this supposition. We found MRP2 expression was decreased by IL-18 treatment at both the mRNA and protein level and in a dose- and time-dependent manner. In BDL rats, we also detected a significantly elevated IL-18 mRNA expression and repressed MRP2 mRNA expression, which were confirmed to be significantly related by linear regression analysis.

So the next question is which transcription factor mediates this down-regulation of the MRP2 gene. It is already known that MRP2 is directly regulated by several nuclear receptors such as FXR, PXR, CAR, and RAR [31]. All these nuclear receptors enable positive regulation to promote the expression of MRP2. No transcription factor has yet been found to suppress MRP2 expression. So the most likely explanation is that IL-18 causes a reduction of a certain nuclear receptor that promotes MRP2 expression and thus, leads to the down-regulation of MRP2. FXR is a nuclear receptor that plays a key role in maintaining cholesterol and bile acid homeostasis [32], and is also a major transcriptional factor that regulates lipid and glucose metabolism in the liver. FXR has been proven to be an important nuclear factor that promotes the expression of MRP2 [31]. We detected the expression of several nuclear receptors and found that the expression of FXR was significantly repressed in HepG2 cells treated with IL-18. Decreased expression of FXR was also observed in BDL rats. This is an interesting finding, because the bile acids chenodeoxycholic acid, lithocholic acid, and deoxycholic acid are ligands for FXR [33]. Therefore, FXR activity should be up-regulated in cholestasis because of the increased bile acids. However, our data indicates that FXR expression is down-regulated in cholestasis at both the mRNA and protein level, which may explain why MRP2 is down-regulated in cholestasis while FXR activity is likely up-regulated. Meanwhile, Cyp7a1 expression was shown to be elevated by IL-18 in HepG2 cells (S1 Fig), which further confirmed that the target genes of FXR could be regulated not only by the activation of FXR, which occurs mainly via ligand binding, but also by the expression level of FXR. ChIP assays further verified that FXR bound to the promoter region in the MRP2 gene was significantly less in liver extracts from the BDL rats than the sham-operated rats. This result further confirms that the reduction of MRP2 in the BDL rats is very likely to be associated with the reduction of FXR. Furthermore, these findings indicate that there must be other inhibitive factors which repress FXR expression. One candidate is the transcription factor YY1.

YY1 is an ubiquitous and multifunctional zinc-finger transcription factor of the polycomb group protein family, which can act as a transcriptional repressor, activator, or initiator element-binding protein [34]. A previous study demonstrated that YY1 can suppress FXR expression by binding to the YY1 responsive element in intron 1 of the FXR gene [23]. We detected the expression of YY1 in HepG2 cells with IL-18 treatment and found that YY1 expression was elevated by IL-18 in a dose-dependent manner at both the mRNA and protein level. In the BDL rats, YY1 expression was also observed to be elevated and closely related to IL-18 expression. To investigate whether down-regulation of FXR and MRP2 were induced by YY1, we transfected HepG2 cells with YY1 shRNA and then treated the cells with IL-18. We discovered that FXR and MRP2 expression were still reduced, but the fold reduction was significantly lower than in normal HepG2 cells. This suggested that the IL-18-mediated decrease of MRP2 and FXR was attenuated by YY1 interference. In the BDL rats, we observed that YY1 mRNA expression was significantly negatively correlated with FXR mRNA expression. These data demonstrate that YY1 expression is enhanced in HepG2 cells with IL-18 treatment, and FXR expression is inhibited by YY1, which then leads to the down-regulation of MRP2.

We then attempted to demonstrate the mechanism by which IL-18 induces YY1 expression. NF-κB is a primary signaling pathway conducted by IL-18 [25], and has been shown in hepatocytes to be activated by IL-18 [35,36]. Upon binding to the IL-18 receptor (IL-18R), IL-18 initiates a signaling cascade that results in the activation of NF-κB [37]. Furthermore, YY1 expression has been shown to be enhanced by NF-κB, which directly binds to the YY1 promoter using its p50/p65 heterodimer [24]. Blockade of NF-κB results in the inhibition of YY1 expression [38]. In our study, NF-κB was confirmed to be activated by IL-18 in HepG2 cells. In the BDL rats, NF-κB was also activated.

To study whether IL-18 repression of MRP2 expression is mediated by the NF-κB pathway, we transfected HepG2 cells with p65 shRNA and then treated the p65 knockdown HepG2 cells with IL-18. We observed that the elevated YY1 expression and repressed MRP2 and FXR levels (i.e., conditions induced by IL-18) were all impaired in the p65 knockdown HepG2 cells. These results reveal that IL-18 can enhance YY1 expression and inhibit FXR and MRP2 expression, and that this regulation is partially mediated by activation of the NF-κB pathway.

## Conclusions

**Fig 7:**
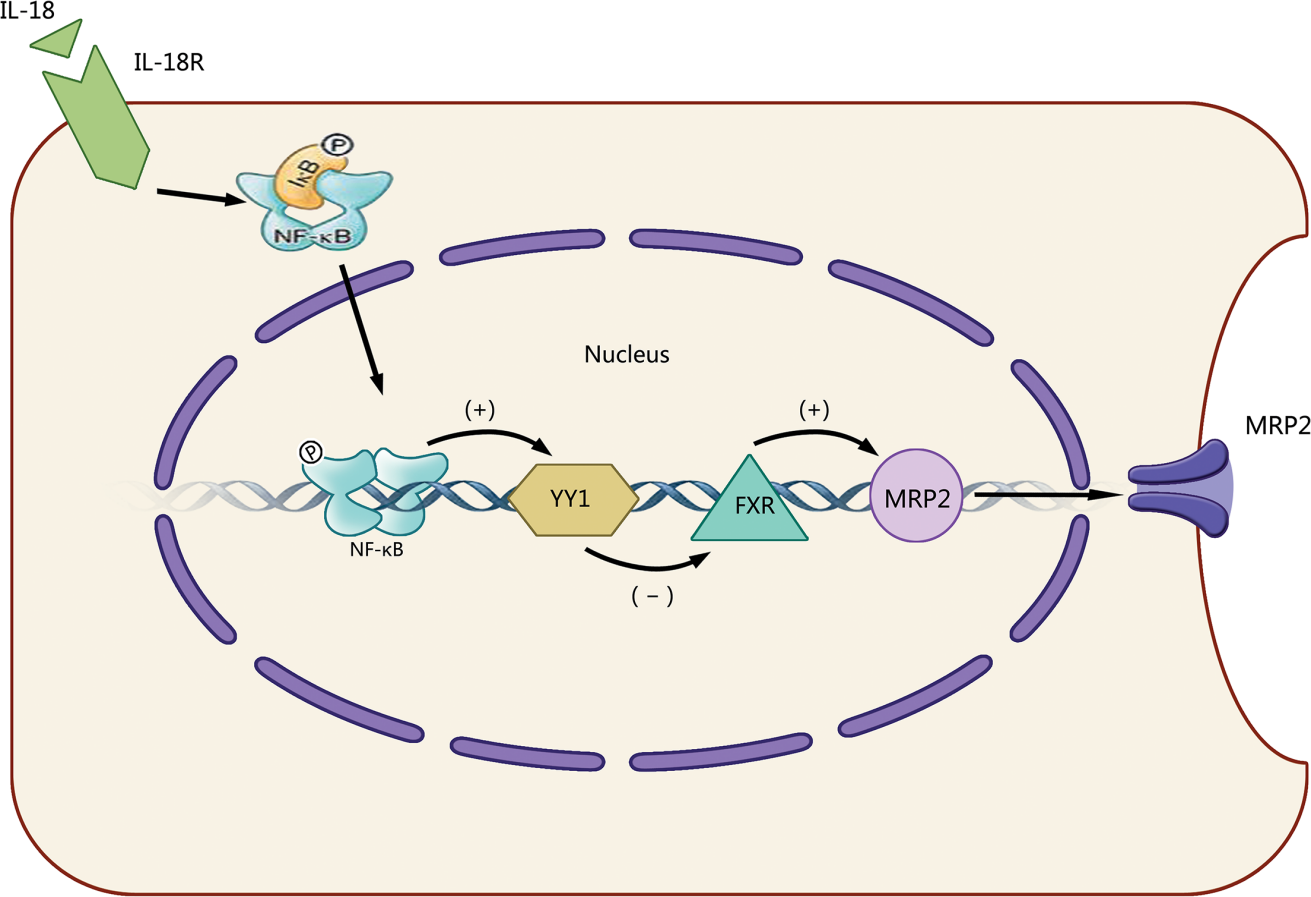
Model of the mechanism of IL-18 induced reduction in MRP2 expression. When HepG2 cells were treated with IL-18, NF-κB is activated, which in turn increases transcription factor YY1 expression; enhanced YY1 decreases FXR expression, then leads to the reduction of MRP2 expression. (+), activation; (-), inhibition; IL-18R, IL-18 receptor; IκB, I kappa B; Ⓟ, phosphorylation.

In summary, our data demonstrate that IL-18 can up-regulate the expression of YY1 through the NF-κB signaling pathway. We suggest a model where elevated YY1 expression suppresses FXR expression, leading to the decrease of MRP2 expression (Fig 7). Our findings represent a novel negative regulation of MRP2, which may contribute to increased understanding of the decreased expression of MRP2 in cholestasis and may also provide new directions to explore in the development of novel therapeutic strategies for clinical treatment of cholestasis.

## Supporting Information

S1 Fig Cyp7a1 expression was elevated by IL-18 in HepG2 cells. IL-18 (30ng/ml) increased the protein expression of Cyp7a1 in HepG2 cells by western blot analysis. Data is given as the mean ± SD (n = 3). *P<0.05.
(TIF)

S1 Table The ARRIVE guidelines checklist. (PDF)
