# Supplementary figures and images for: Interleukin-18 Down-Regulates Multidrug Resistance-Associated Protein 2 Expression through Farnesoid X Receptor Associated with Nuclear Factor Kappa B and Yin Yang 1 in Human Hepatoma HepG2 Cells
Source: PLoS One. 2015 Aug 20;10(8):e0136215. doi: 10.1371/journal.pone.0136215 (PMC4546195; doi:10.1371/journal.pone.0136215)

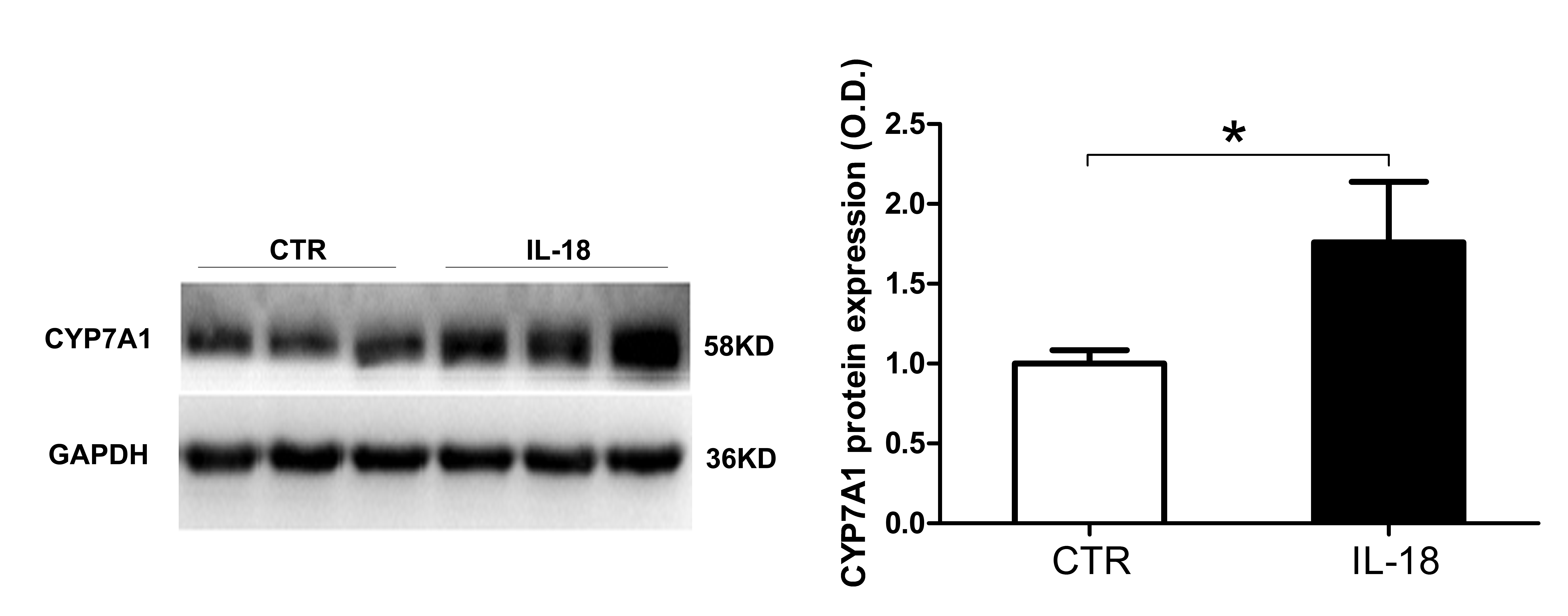

Supplement: S1 Fig — IL-18 (30ng/ml) increased the protein expression of Cyp7a1 in HepG2 cells by western blot analysis. Data is given as the mean ± SD (n = 3). *P<0.05. (TIF) [file pone.0136215.s001.tif]
